# Development of an Undifferentiated Pleomorphic Sarcoma After Aortic Aneurysm Graft Replacement: A Case Report and Literature Review

**DOI:** 10.7759/cureus.61530

**Published:** 2024-06-02

**Authors:** Yotaro Asano, Aoi Utsunomiya, Shiori Meguro, Masaki Sano, Kazunori Inuzuka, Hiroya Takeuchi, Hideya Kawasaki, Isao Kosugi, Yasunori Enomoto, Mayu Fujihiro, Satoshi Baba, Toshihide Iwashita

**Affiliations:** 1 Department of Regenerative and Infectious Pathology, Hamamatsu University School of Medicine, Hamamatsu, JPN; 2 Department of Surgery, Hamamatsu University School of Medicine, Hamamatsu, JPN; 3 Institute for NanoSuit Research, Preeminent Medical Photonics Education & Research Center, Hamamatsu University School of Medicine, Hamamatsu, JPN; 4 Department of Diagnostic Pathology, Hamamatsu University School of Medicine, Hamamatsu, JPN; 5 Department of Diagnostic Pathology, Hamamatsu University Hospital, Hamamatsu, JPN

**Keywords:** histopathology, post-mortem diagnosis, undifferentiated pleomorphic sarcoma, sarcoma, aortic graft, autopsy

## Abstract

Aortic sarcomas are extremely rare. Sarcomas associated with aortic graft replacement are even rarer; only 17 cases have been examined through immunohistochemical staining to date, most of which were either angiosarcomas or intimal sarcomas. Here, we report the case of an 88-year-old man with an undifferentiated pleomorphic sarcoma (UPS) that developed after aortic graft replacement and was diagnosed through postmortem autopsy. To the best of our knowledge, this is the first case of graft-associated sarcoma diagnosed as an undifferentiated pleomorphic type following detailed immunohistochemical staining with sufficient antibodies and fluorescencein situ hybridization (FISH).

## Introduction

Primary aortic sarcoma is a rare disease, with approximately 190 reported cases [1]. Its low incidence and unclear definition make its diagnosis difficult for pathologists and clinicians. Two-thirds of all aortic sarcomas arise from the intima (intimal-type sarcomas); the remaining arise from the tunica media or adventitia (mural-type sarcomas). Intimal-type sarcomas include angiosarcomas and intimal sarcomas, whereas mural-type sarcomas include leiomyosarcomas and undifferentiated pleomorphic sarcomas (UPSs) [2-3].

Sarcomas associated with aortic grafts used in aneurysm repair are extremely rare, with only 17 case reports involving immunostaining analysis available in the Medical Literature Analysis and Retrieval System Online (MEDLINE) database [4]. Most of these cases involved intimal-type sarcomas (angiosarcomas and intimal sarcomas). To the best of our knowledge, no reports of an aortic graft-associated sarcoma histologically confirmed as a UPS using sufficient antibodies and fluorescence in situ hybridization (FISH) currently exist. Herein, we describe a case of a UPS associated with aortic graft replacement that was confirmed through a postmortem autopsy. In addition, we have summarized cases of aortic graft-associated sarcomas according to their immunostaining findings and histological types [5-20].

## Case presentation

The patient was an 88-year-old man. Fifty-nine months before his death, he underwent aortic graft replacement through open surgery with a prosthetic vascular Y-graft for an abdominal aortic aneurysm distal to the renal artery bifurcation (maximum diameter, 50 mm). The Y-graft was constructed from Dacron fibers knitted into a cylindrical shape and coated on the surface with collagen extracted from bovine tendons (InterGard K 20 × 10 mm, MAQUET Japan K.K., Tokyo, Japan). Postoperative follow-up CT scans were conducted to detect anastomotic leakage or pseudoaneurysm after the aortic graft replacement.

**Figure 1:**
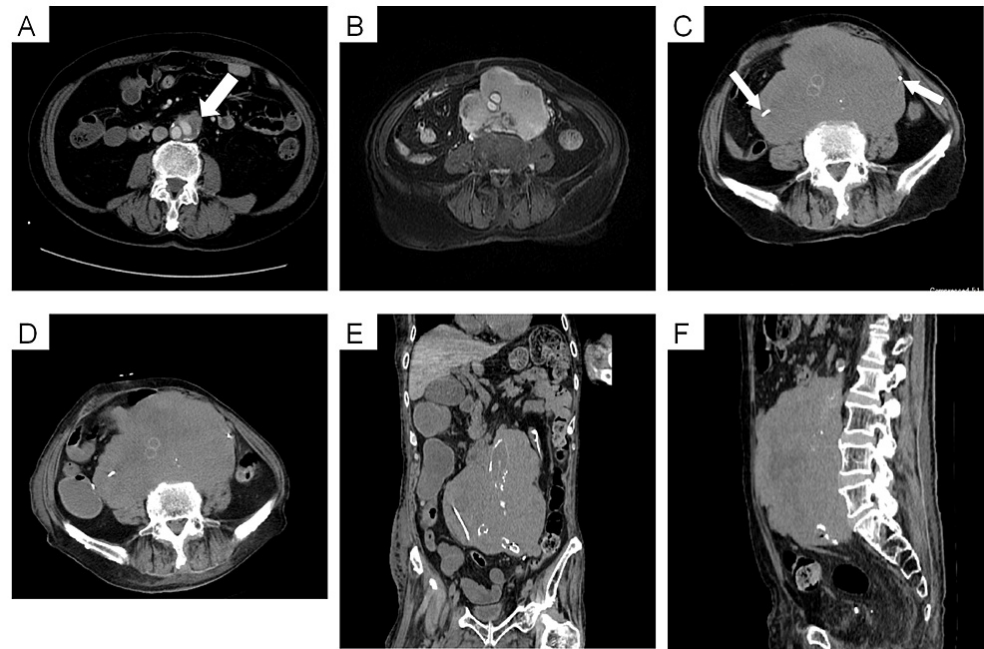
Imaging findings over time (A) Contrast-enhanced computed tomography (CECT) image acquired 22 months before death (i.e., 37 months postoperatively). The arrow shows a seroma-like, low-density area around the Y-shaped graft in the abdominal aorta and common iliac arteries. (B) A contrast-enhanced, T1-weighted magnetic resonance image obtained 15 months before death (i.e., 44 months postoperatively) shows that the mass increased to approximately 10 × 9 cm. (C) A simple CT scan obtained five months before death (i.e., 54 months postoperatively) shows further enlargement of the mass. The arrows indicate the site of ureteral stent replacement. (D, E, and F) Simple CT scans obtained 59 months postoperatively show the horizontal, coronary, and sagittal sections, respectively. A mass, measuring approximately 17 × 16 × 12 cm in each section, is noted in the retroperitoneum.

During the follow-up conducted 22 months before the patient’s death (i.e., approximately 37 months postoperatively), the clinician observed a low-density mass in the retroperitoneum around the aortic graft, which was diagnosed as a seroma via computed tomography (Figure 1A). Doppler echocardiography revealed no aortic leakage. At 15 months before death (i.e., 44 months postoperatively), the mass had increased to approximately 10 × 9 cm (Figure 1B). The clinician considered the lesion to be neoplastic [A1] rather than an inflammatory disease, such as IgG4-related sclerosing disease. Regarding the neoplastic lesion, they included an aortic graft-associated sarcoma and retroperitoneal tumor in the differential diagnosis. Invasive treatments, such as surgical resection and chemotherapy, were considered difficult given the patient’s advanced age and poor general condition. Five months before the patient’s death (i.e., 54 months postoperatively; Figure 1C), bilateral ureteral stents were placed because the masses compressed the bilateral ureters and caused hydronephrosis. Shortly before the patient's death (i.e., 59 months postoperatively; Figures 1D, 1F), he was admitted to our hospital due to worsening respiratory status caused by pneumonia in the right upper and middle lobes. His renal function deteriorated (potassium, 6.6 mEq/L; blood urea nitrogen, 67.5 mg/dL; creatinine, 5.1 mg/dL) due to bilateral ureteral compression caused by the increasing mass, as well as respiratory failure. The patient eventually died due to multi-organ failure. After death, a pathological autopsy was performed with the consent of the patient’s family.

**Figure 2:**
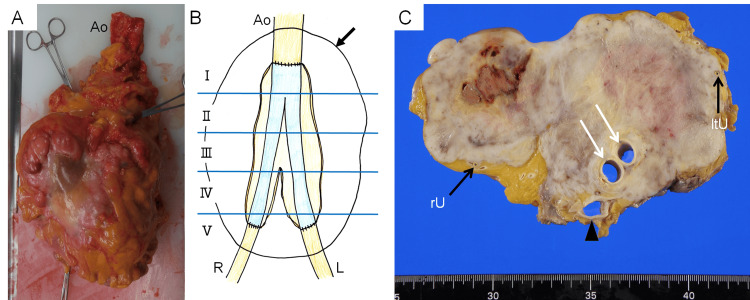
Macroscopic details of the tumor (A) The mass (2470 g) surrounding the Y-graft in the abdominal aorta and common iliac arteries. Ao indicates the aorta. (B) A schematic illustration of Figure A showing the mass, aorta, common iliac arteries, and Y-graft (blue). The mass is divided almost equally into five sections named Ⅰ through Ⅴ. The arrow indicates the outline of the mass. Ao, R, and L indicate the aorta, right common iliac artery, and left common iliac artery, respectively. (C) Cross-section of the mass surrounding the Y-graft (white arrows) in section Ⅱ. The black arrows indicate the ureters. The inferior vena cava is open (black arrowhead). rU and ltU indicate the right ureter and left ureter, respectively. Figure 2B has been created by Masaki Sano and Kazunori Inuzuka.

Macroscopically, a 17 × 16 × 12 cm yellowish-white solid mass surrounded the Y-graft below the renal artery bifurcation (Figures 2A, 2B). However, no occlusion or stenosis of the Y-graft was observed, and the Y-graft lumen was smooth (Figure 2C); thus, a neoplastic lesion arising from the intima was ruled out. The right and left ureters were completely surrounded by the mass and were compressed and narrowed from the outside.

**Figure 3:**
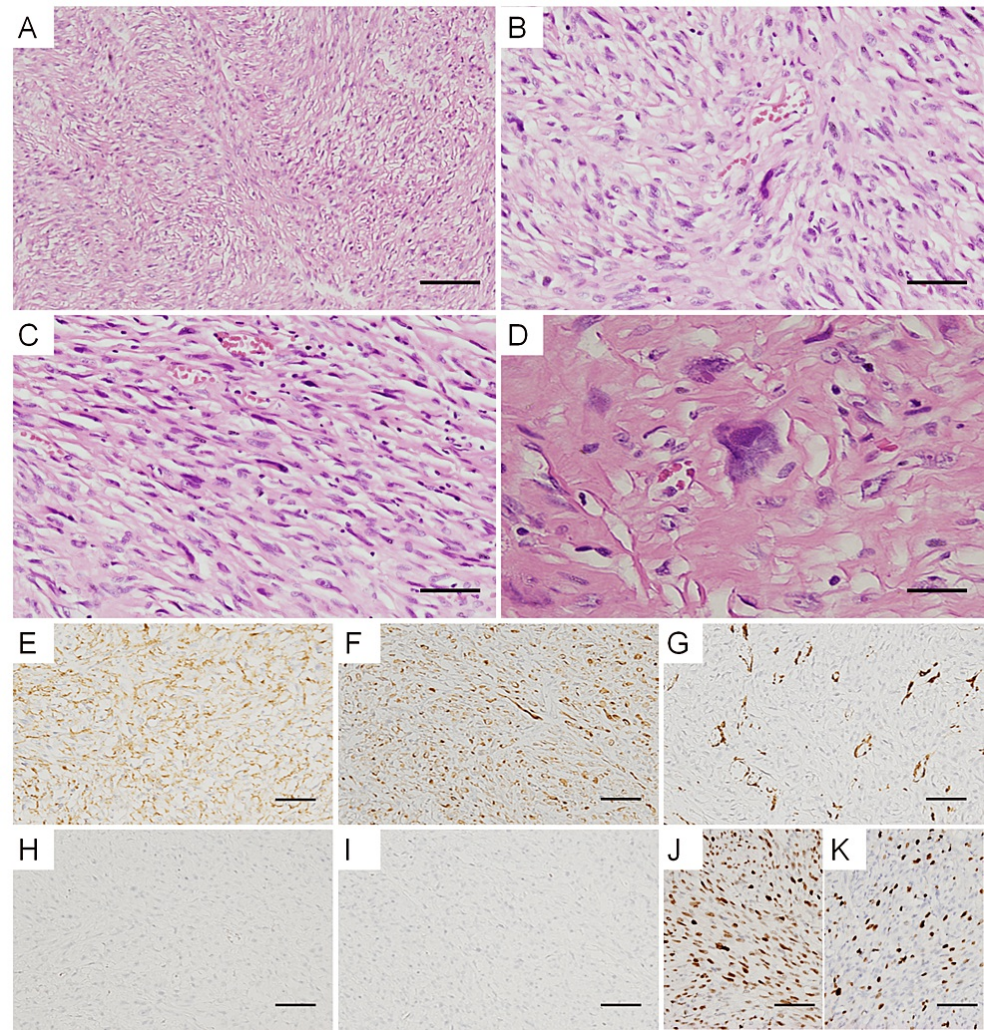
Histology and immunostaining for the tumor (A, B) Hematoxylin and eosin staining of the tumor. (A) The proliferation of spindle-shaped and atypical cells is observed, and a partially storiform pattern is visible (×100). (B, C) Nuclear atypia is observed (×200). (D) Prominent nucleoli and bizarre nuclei are scattered throughout (×400). (E) CD68, (F) AE1/AE3, (G) α-SMA, (H) CDK4, (I) MDM2, (J) p53, and (K) Ki-67 staining of the tumor (×100). The tumor cells are positive for CD68, AE1/AE3, and p53. Scale bars = 100 µm (A, E, F, G, H, I, J, and K), 50 µm (B, C), and 25 µm (D).

Histologically, a dense proliferation of spindle-shaped cells partially arranged in a storiform manner was noted (Figure 3A). The proliferating cells were highly atypical, with prominent nucleoli (Figures 3B-3D). Abnormal mitotic figures were not observed; the number of mitotic figures per 10 high-power fields was approximately one to two. Large, multinucleated giant cells and cells with unusual nuclei were scattered within the mass. The tumor cells had grown to encircle the aortic graft and had mildly infiltrated the tunica media and intima from the adventitia side.

Immunohistochemically, the tumor cells obtained were positive for vimentin, CD68 (Figure 3E), and AE1/AE3 (Figure 3F) and negative for S100, SOX10, desmin, α-SMA (Figure 3G), h-caldesmon, calponin, HHF35, MyoD1, myogenin, CD31, CD34, ERG, BCL-2, and TLE-1. This indicated that the tumor cells did not differentiate into neuronal cells, neural crest cells, smooth muscle cells, myofibroblasts, skeletal muscle cells, or vascular endothelial cells. Moreover, the tumor cells were negative for STAT6 and beta-catenin; thus, a solitary fibrous tumor and a desmoid tumor were ruled out. The tumor cells were also immunohistochemically negative for CDK4 (Figure 3H) and MDM2 (Figure 3I), and FISH revealed no amplification of MDM2 within the cells; thus, a liposarcoma was ruled out. The tumor cells were immunohistochemically positive for H3K27me3a; thus, a malignant peripheral nerve sheath tumor was ruled out. Additionally, p53 was diffusely positive (Figure 3J), and Ki-67 was positive in approximately 30% of the tumor cells (Figure 3K); these findings suggested high-grade malignancy. Accordingly, the tumor was diagnosed as a UPS. The autopsy results showed no evidence of tumor metastasis to other organs.

## Discussion

We present a case of a UPS that developed as a large mass around an aortic graft and only mildly invaded the aortic media and intima. Therefore, the sarcoma may not have originated from the aortic intima or tunica media but from the aortic adventitia or retroperitoneum surrounding the aorta. Among the 29 cases of graft-associated sarcomas reported to date, immunochemical staining was performed in 17 cases (Table 1).

**Table 1:** Histological and immunohistochemical features of sarcomas associated with aortic grafts AS: angiosarcoma; eAS: epithelioid angiosarcoma; IS: intimal sarcoma; SPS: spindle and pleomorphic sarcoma; LS: leiomyosarcoma; UPS: undifferentiated pleomorphic sarcoma; P: positive; N: negative; FISH: fluorescence in situ hybridization; AR: aortic replacement with prosthetic graft; EVAR: endovascular aortic repair

Author, year of publication	Histological diagnosis	Immunohistochemical findings	Surgical procedures
Weiss et al., 1991 [5]	AS	P: vimentin, factor Ⅷ; N: cytokeratins	AR
Ben­Izhak et al., 1991 [6]	eAS	P: CD31, CD34, factor Ⅷ, AE1/AE3, CAM5.2	AR
Okada et al., 2004 [7]	AS	P: CD31; N: CD34, factor Ⅷ	AR
Umscheid et al., 2007 [8]	eAS	P: factor Ⅷ, CD34; N: cytokeratins	EVAR
Alexander et al., 2007 [9]	IS	P: vimentin, pancytokeratin; N: CD31, CD34, factor Ⅷ, desmin, SMA, cytokeratin 8/18, EMA	AR
Garg et al., 2012 [10]	IS	P: CD31, Fil-1, CK7; N: CK20	EVAR
Schmehl et al., 2012 [11]	eAS	P: CD31, pancytokeratin, p53 (10%); N: CD30, EMA, CD34	EVAR
Stewart et al., 2013 [12]	IS	P: vimentin, CD31; N: CD34, desmin, SMA, pancytokeratin	EVAR
Milite et al., 2016 [13]	eAS	P: vimentin, CD31, CD34, factor Ⅷ	EVAR
Kamran et al., 2016 [14]	AS	P: vimentin, CD31	EVAR
Whittington et al., 2019 [15]	SPS	P: CD68	EVAR
Natsume et al., 2019 [16]	IS	P: CD31, MDM2	EVAR
Presacco et al., 2020 [17]	AS	P: CD31, ERG, FLI-1	EVAR
Derouane et al., 2020 [18]	eAS	P: CD31, ERG	EVAR
Sultan et al., 2020 [19]	LS	P: vimentin, SMA N: c-Kit, S100, CD31, CD99, CD34.	EVAR
Takamura et al., 2021 [4]	eAS	P: vimentin, CD31, factor Ⅷ, ERG, pancytokeratin, CDK4; N: MDM2	EVAR
Komatsu et al., 2022 [20]	IS	P: CD31, MDM2	EVAR
Our case	UPS	P: vimentin, CD68, AE1/AE3, p53, H3K27me3a N: SMA, h-caldesmon, calponin, HHF35, MyoD1, myogenin, CD31, ERG, BCL-2, TLE-1, STAT6, CDK4, MDM2 (immunohistochemistry and FISH), CD34, desmin, beta-catenin	AR

The most common histological type was angiosarcoma (n = 10), followed by intimal sarcoma (n = 5). Thus, intimal-type sarcomas accounted for 15 of the 17 cases of graft-associated sarcomas; mural-type sarcomas accounted for the remaining two cases. In one of these two cases, the histological characteristics of the primary lesion around the aorta were unknown; however, hematoxylin and eosin staining of the metastatic liver tissue indicated a pleomorphic sarcoma. Furthermore, the authors did not mention any immunostaining findings, except that the sarcoma was CD68 positive; this made it difficult to determine the final histopathological diagnosis [15]. In the other case, immunostaining revealed that the tumor cells were positive for vimentin and α-SMA, while negative for c-Kit, S100, CD31, CD99, and CD34; the final pathological diagnosis was leiomyosarcoma [19].

With hematoxylin and eosin staining, UPSs are often difficult to distinguish from other sarcomas that show cell pleomorphism; therefore, immunohistochemical staining with sufficient antibodies or FISH is used to identify them. In the present case, the tumor was considered unlikely to be a leiomyosarcoma, rhabdomyosarcoma, peripheral malignant nerve sheath tumor, or liposarcoma on the basis of immunohistochemical and FISH findings. Based on these results, the tumor was diagnosed as a UPS.

## Conclusions

Aortic sarcomas are exceedingly rare. There have been only 17 cases of sarcomas associated with aortic graft replacement that have been analyzed using immunohistochemical staining so far. The majority of these cases were either angiosarcomas or intimal sarcomas. In this case report, we described an 88-year-old man with a UPS that developed after aortic graft replacement and was diagnosed through a postmortem autopsy. To the best of our knowledge, this is the first case of graft-associated sarcoma diagnosed as a UPS following a detailed histopathological examination. 
